# *De-novo* emergence of SINE retroposons during the early evolution of passerine birds

**DOI:** 10.1186/s13100-017-0104-1

**Published:** 2017-12-14

**Authors:** Alexander Suh, Sandra Bachg, Stephen Donnellan, Leo Joseph, Jürgen Brosius, Jan Ole Kriegs, Jürgen Schmitz

**Affiliations:** 1 Institute of Experimental Pathology (ZMBE), University of Münster, D-48149 Münster, Germany; 2 Department of Evolutionary Biology (EBC), Uppsala University, SE-75236 Uppsala, Sweden; 3 South Australian Museum, Adelaide, SA 5000 Australia; 4 School of Biological Sciences, The University of Adelaide, Adelaide, 5005 Australia; 5 Australian National Wildlife Collection, CSIRO National Research Collections Australia, Canberra, ACT 2601 Australia; 6 Brandenburg Medical School (MHB), D-16816 Neuruppin, Germany; 7 LWL-Museum für Naturkunde, Westfälisches Landesmuseum mit Planetarium, D-48161 Münster, Germany

**Keywords:** Transposon, Retroposon, SINE, Birds, Passeriformes, Phylogenomics

## Abstract

**Background:**

Passeriformes (“perching birds” or passerines) make up more than half of all extant bird species. The genome of the zebra finch, a passerine model organism for vocal learning, was noted previously to contain thousands of short interspersed elements (SINEs), a group of retroposons that is abundant in mammalian genomes but considered largely inactive in avian genomes.

**Results:**

Here we resolve the deep phylogenetic relationships of passerines using presence/absence patterns of SINEs. The resultant retroposon-based phylogeny provides a powerful and independent corroboration of previous sequence-based analyses. Notably, SINE activity began in the common ancestor of Eupasseres (passerines excluding the New Zealand wrens Acanthisittidae) and ceased before the rapid diversification of oscine passerines (suborder Passeri – songbirds). Furthermore, we find evidence for very recent SINE activity within suboscine passerines (suborder Tyranni), following the emergence of a SINE via acquisition of a different tRNA head as we suggest through template switching.

**Conclusions:**

We propose that the early evolution of passerines was unusual among birds in that it was accompanied by *de-novo* emergence and activity of SINEs. Their genomic and transcriptomic impact warrants further study in the light of the massive diversification of passerines.

**Electronic supplementary material:**

The online version of this article (10.1186/s13100-017-0104-1) contains supplementary material, which is available to authorized users.

## Background

Short interspersed elements (SINEs) are the most abundant group of the reverse-transcribed retroposons in mammalian genomes [1]. They rely on *trans*-mobilization by the enzymatic machinery of long interspersed elements (LINEs) [2], a *parasitic* interaction so successful that the human genome contains >1,500,000 SINEs compared to <900,000 LINEs [3]. On the other hand, SINEs are scarce in avian genomes, and this has been noted as one of the most peculiar genomic features of birds [4–6]. While LINEs exhibit up to 700,000 copies in avian genomes, there are only 6000–17,000 SINEs per avian genome [6], most of these being ancient and heavily degraded [7].

Presence/absence patterns of SINEs in orthologous genomic loci are rare genomic changes appreciated widely as virtually homoplasy-free phylogenetic markers [8, 9]. Given the aforementioned scarcity of SINEs, it is not surprising that the emergence and activity of SINEs has never been studied in birds. On the other hand, other types of retroposed elements (REs; LINEs from the chicken repeat 1 superfamily, CR1, and long terminal repeat elements, LTRs) have helped resolve the relationships of various groups of birds, such as Galliformes [10–12], Neoaves [13–15], Palaeognathae [16, 17], and others [18–21]. In the meantime, the sequencing of dozens of avian genomes has revealed SINEs with putative lineage specificity [5, 7, 22] and thus the potential for conducting phylogenetic presence/absence analyses in specific groups of birds.

Here we conduct, to our knowledge, the first study of the emergence and activity of SINEs in birds. We focus on the deep phylogenetic relationships of passerines, the largest radiation of birds with nearly 6000 extant species [23], using 44 presence/absence markers of SINEs and other REs. In contrast to the only previous study of retroposons in passerines with a single RE marker [24], our multilocus dataset permits the reassessment of sequence-based phylogenies (e.g., [23, 25, 26]) and, simultaneously, the reconstruction of the temporal activity of SINEs and other REs during early passerine evolution.

## Results and discussion

### Two CR1-mobilized SINEs in passerines

We initially chose RE marker candidates from selected retroposon families of the oscine passerine zebra finch *Taeniopygia guttata* (including TguSINE1, [5]; Additional file 1: Table S1) in October 2009, a time when genome assemblies were available only for chicken and zebra finch [4, 5]. Seventy four candidates for presence/absence loci were therefore identified via pairwise alignment of RE-flanking sequences from zebra finch to orthologous regions in chicken (Materials and Methods). This was followed by in-vitro presence/absence screening of RE marker candidates as detailed elsewhere [13, 27] using a representative taxon sampling of all major groups of passerines sensu Barker et al. [23] (Additional file 1: Table S2). We complemented this with a screening of GenBank [28] for additional SINEs, which identified a TguSINE1-like insertion in *myoglobin* intron 2 of the suboscine *Pitta anerythra* (accession number DQ785977) that is absent in the orthologous position of other *Pitta* species [29]. We termed this element “PittSINE” and identified PittSINE marker candidates in a DNA sample of *Pitta sordida* via inter-SINE PCR ([30]; Methods). This was followed by cloning of the 500-bp to 1000-bp fraction of PCR amplicons and sequencing of 24 clones, alignment to chicken and zebra finch genomes to reconstruct the left and right SINE-flanking regions, and then in-vitro presence/absence screening of nine PittSINE marker candidates.

**Fig. 1:**
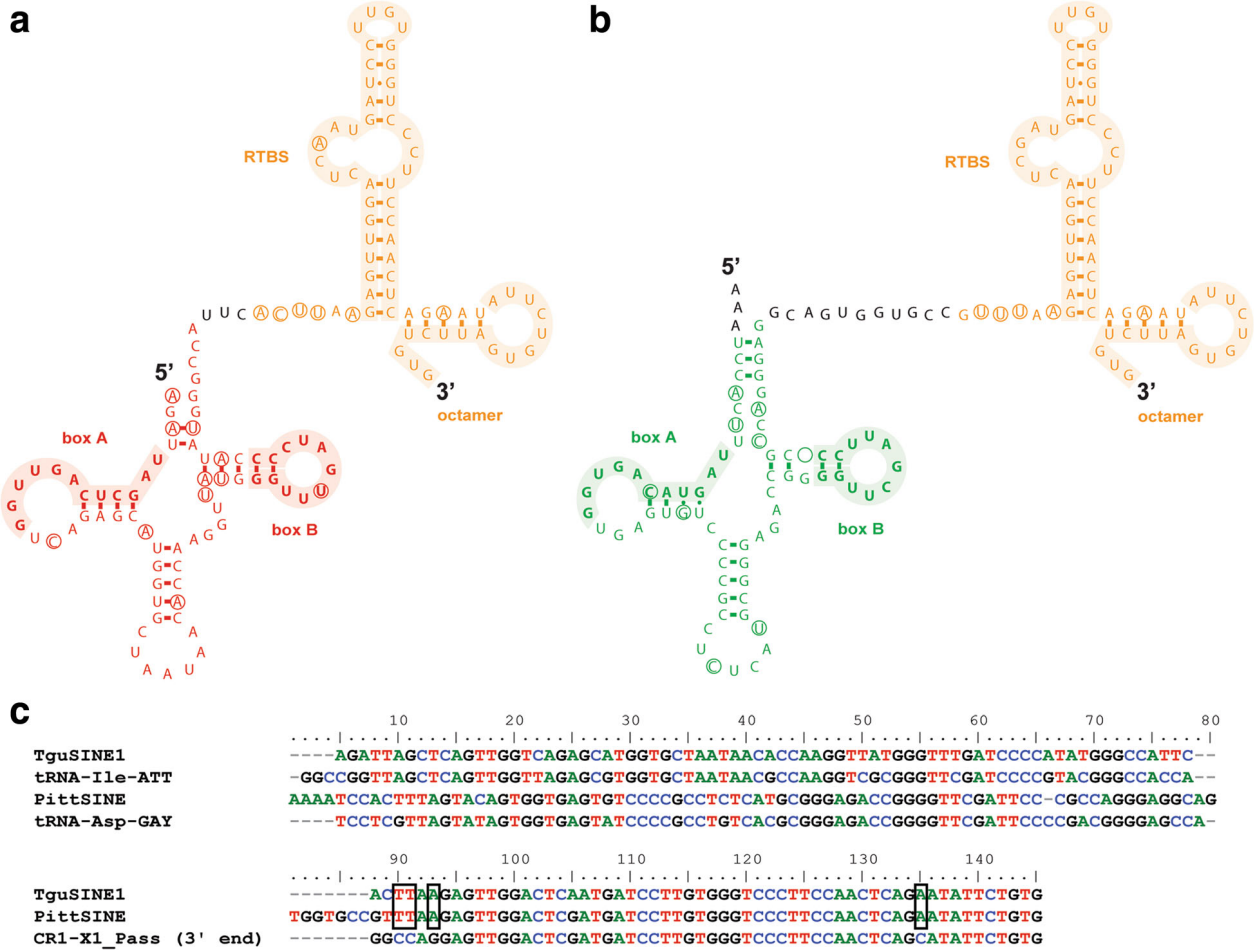
Proposed RNA secondary structures of passerine SINEs with CR1-derived tails (orange) and tRNA-derived heads. The SINE heads are tRNA^Ile^ (red) in TguSINE1 (**a**) and tRNA^Asp^ (green) in PittSINE (**b**). Shaded regions denote promoter boxes A and B in tRNAs, as well as the reverse transcriptase binding site (RTBS) and 5′-AUUCURUG-3′ microsatellite typical for CR1 elements of amniotes [31]. Circles indicate nucleotide differences between SINE consensus sequences and the respective tRNAs or CR1 they are derived from. The RTBS hairpin structure is also visible in mfold [57] predictions of SINE secondary structure (Additional file 3: Figure S1). **c** DNA sequence alignment of TguSINE1 and PittSINE with respective tRNA genes and the 3′ end of CR1-X1_Pass. Black boxes denote diagnostic nucleotides present in the CR1-derived tails of TguSINE1 and PittSINE

Next, we characterized the structural organization of passerine SINEs (Fig. 1) using the available TguSINE1 consensus sequence [5] and after generating a majority-rule consensus of six PittSINE insertions in our sequenced presence/absence markers (Additional file 2). Both SINEs have highly similar, CR1-derived tails (Fig. 1) which exhibit the typical hairpin for putative binding by the CR1 reverse transcriptase and an 8-bp microsatellite at their very end for target-primed reverse transcription [31] (Additional file 3: Figure S1). However, the heads of these SINEs are derived from different tRNA genes, namely tRNA^Ile^ in TguSINE1 and tRNA^Asp^ in PittSINE (Fig. 1). Sequence alignment suggests that the tRNA-derived SINE heads are more similar to the respective tRNA genes than they are to each other (Fig. 1c). However, the opposite is the case for the CR1-derived SINE tails, which exhibit four diagnostic nucleotides distinguishing them from the highly similar 3′ end of CR1-X1_Pass (Fig. 1c). To verify that these are specific to TguSINE1 and PittSINE, we screened the zebra finch genome assembly for the presence of the four diagnostic nucleotides in copies of CR1-X1_Pass. Among those copies most similar to CR1-X1_Pass, only one old copy (chr2:68,921,881–68,922,556) contained the four diagnostic nucleotides, suggesting that these were acquired randomly after the insertion event.

**Fig. 2:**
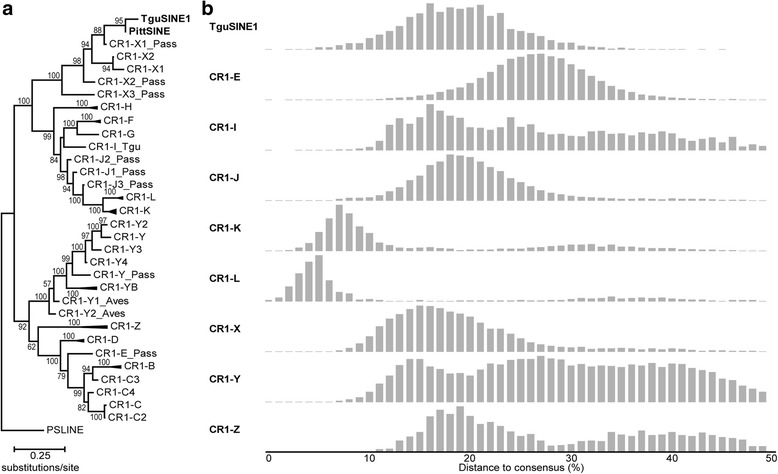
Passerine SINEs share a common ancestor and are mobilized by CR1-X. **a** Maximum likelihood phylogeny of passerine SINE tails and avian CR1 subfamilies in Repbase [58] (GTRCAT model, 1000 bootstrap replicates) suggests that TguSINE1 and PittSINE arose from the same CR1-X subfamily (CR1-X1_Pass) and share a common SINE ancestor. Note that the topology of the CR1 phylogeny is identical to that of previous studies [20, 32]. **b** Comparison of the TguSINE1 landscape with landscapes of CR1 families (merged subfamilies from panel A) suggests temporal overlap of TguSINE1 and CR1-X activity in the zebra finch genome. RE landscapes were generated using the zebra finch assembly taeGut2 following methods detailed elsewhere [32]

**Fig. 3:**
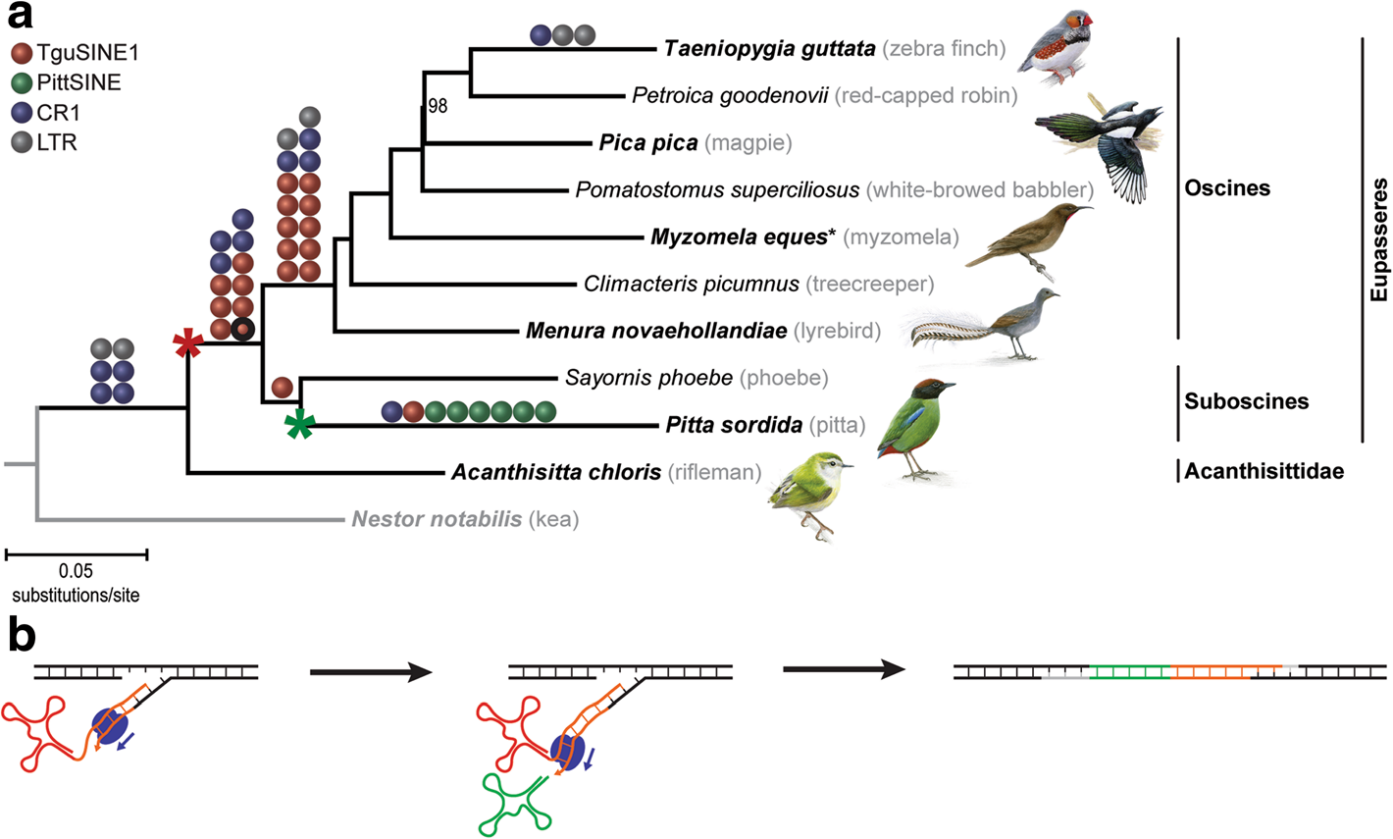
Emergence and timing of CR1-mobilized SINE activity during early passerine evolution. **a** Phylogenomic analysis of early passerine relationships using retroposon presence/absence markers (colored balls) mapped on a maximum likelihood phylogeny of concatenated retroposon-flanking sequences (GTRCAT model, 1000 bootstrap replicates; Additional file 5). The single conflicting marker on the Eupasseres branch (Tgu10, cf. Additional file 1: Table S2) is indicated by a red ball with black circle and was likely affected by incomplete lineage sorting within Suboscines. Our sampling consists of the major deep passerine lineages sensu Barker et al. [23]. The later additions of two genome assemblies (*Corvus cornix* and *Manacus vitellinus*) were only included in the presence/absence table (Additional file 1: Table S2). Red and green asterisks indicate emergence of TguSINE1 and PittSINE, respectively. The black asterisk indicates that for some loci (Additional file 1: Table S2), *Malurus cyaneus* was sampled instead of *Myzomela eques* to represent the Maluridae/Meliphagidae clade [23]. Only bootstrap values <100% are shown and the names of pictured birds are emphasized in bold. **b** A scenario for the emergence of PittSINE. Template switching from TguSINE1 RNA (red, tRNA^Ile^ head; orange, CR1 tail) to tRNA^Asp^ (green) during target-primed reverse transcription by CR1 reverse transcriptase (blue). The resultant tRNA^Asp^-CR1 chimaera was flanked by a target site duplication (grey) and transcriptional activation gave rise to the PittSINE family

We further investigated this peculiar pattern using phylogenetic analyses of the CR1-derived SINE tails and avian CR1 subfamilies sensu ref. [32], which again suggests that TguSINE1 and PittSINE have a single SINE ancestor which derived its tail from CR1-X1_Pass (Fig. 2a). Assuming that SINEs are *trans*-mobilized by LINE reverse transcriptase enzymes due to high sequence similarity between SINE tails and LINE 3′ ends [2, 33] and thus depend on LINE activity, the most likely candidate for SINE mobilization is the CR1-X1_Pass subfamily. This is further supported by temporal overlap of TguSINE1 and CR1-X activity in RE landscapes of the zebra finch genome (Fig. 2b). Additionally, we detected direct evidence for temporal overlap of TguSINE1 and CR1-X1_Pass activity through our presence/absence analyses (Fig. 3a, Additional file 1: Table S2).

### Retroposon-based phylogeny of passerines

**Fig. 4:**
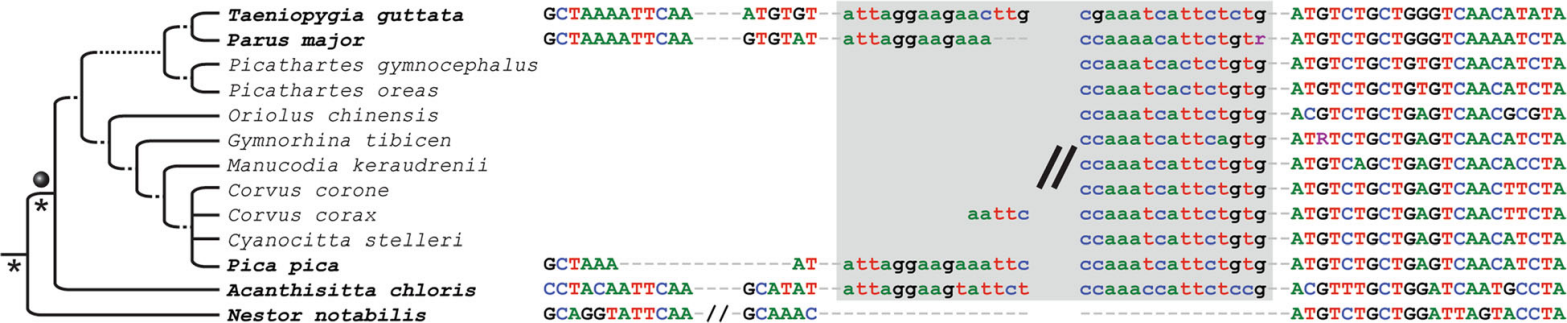
A reassessment of the RE marker of Treplin & Tiedemann [24] through extended taxon sampling. Treplin & Tiedemann [24] inferred “*phylogenetic affinity of rockfowls (genus* Picathartes*) to crows and ravens (Corvidae)*” based on the Cor2 locus which they sequenced in rockfowls and corvids, and unsuccessful Cor2 PCR amplification in other passerines. We generated a nested oligonucleotide primer pair (5′- CAATACTTTGGAACACCTCAGAC-3′ and 5′- GGCACCTGTCAATGGCTAC-3′) and were able to amplify and sequence the Cor2 locus in additional species. Our extended phylogenetic sampling suggests that the RE insertion (lowercase nucleotides) occurred in the ancestor of all passerines (grey ball) due to RE presence in non-corvid passerines (*Taeniopygia guttata*, *Parus major*, *Acanthisitta chloris*) and RE absence in the parrot outgroup (*Nestor notabilis*). Taxa with bold names were sampled in the present study and the grey box denotes the 5′ and 3′ end of the CR1 insertion. Asterisks indicate branches in the avian Tree of Life which were previously recovered with significant support from retroposon markers [13]

Our extensive RE presence/absence analyses yielded 19 TguSINE1, 6 PittSINE, 13 CR1, and 6 LTR markers which we could trace across a representative taxon sampling of the major groups of passerines sensu Barker et al. [23] (cf. [34]). These RE markers are only those where we were able to obtain sequences for all taxa critical for a phylogenetic conclusion. Careful inspection of presence/absence alignments using strict criteria (see Materials and Methods) yielded a conflict-free set of RE markers (except for one marker potentially affected by incomplete lineage sorting; Fig. 3a), which we mapped on a maximum likelihood tree constructed from concatenated RE-flanking sequences from the same data set (Fig. 3a). For three of the deepest passerine branching events, we found a multitude of RE markers and thus statistically significant support in available RE marker tests [35, 36]. These relationships are the respective monophyly of passerines and oscines, as well as the monophyly of Eupasseres [37], a group comprising all passerines except the New Zealand wrens Acanthisittidae. The Eupasseres/Acanthisittidae split was first observed in sequence analyses of few nuclear genes [38, 39] and has since been confirmed in ever-growing nuclear sequence analyses (e.g., [23, 25, 26, 40]). Our analysis of rare genomic changes thus provides the first assessment of this group using an independent marker type and phylogenetic method. None of our RE markers inserted during the rapid radiation of oscine passerines, however, sequence analysis of the RE-flanking regions yielded a topology identical to the aforementioned previous studies. Of particular interest are the four deep-branching oscine lineages Menuridae (e.g., *Menura novaehollandiae*), Climacteridae (e.g., *Climacteris picumnus*), Maluridae/Meliphagidae (e.g., *Malurus cyaneus* and *Myzomela eques*), and Pomatostomidae (e.g., *Pomatostomus superciliosus*) because these four lineages together have been rarely included in passerine phylogenetic studies. We find a branching order (Fig. 3a) which recapitulates previous phylogenetic estimates based on few nuclear genes [23] or ultraconserved elements [26]. This suggests that the rapid radiation of oscines can be congruently resolved even with non-genome-scale data. We note that this is in contrast to the neoavian radiation, which appears to be partially unresolvable even with genome-scale sequence analyses and thousands of retroposon markers (reviewed by [41]). Within passerines, we further note that the conflict between single-RE support for a Picathartidae/Corvidae clade [24] and sequence-based phylogenies [42] results from incorrect placing of this RE marker on the passerine Tree of Life due to methodological limitations (see legend of Fig. 4 for more information).

### Emergence and activity of passerine SINEs

We then traced the emergence and activity of SINEs across the passerine Tree of Life. Given that RE marker candidates were initially chosen on chicken/zebra finch alignments, we expect no bias in the age distribution of RE markers on the lineage leading to zebra finch. TguSINE1 was mostly active in the ancestor of oscines and, to a lesser extent, in the ancestor of Eupasseres. Interestingly, we find no evidence for TguSINE1 activity in the common ancestor of passerines (cf. Additional file 3: Figure S2) or during/after the radiation of oscines and therefore hypothesize that TguSINE1 emerged in Eupasseres and became extinct in the oscines’ ancestor (Fig. 3a). The emergence of TguSINE1 is thus the first synapomorphic “genome morphology” character for Eupasseres and supplements support from skeletal morphology, which is limited to the presence of a ‘six-canal pattern’ in the hypotarsus [43].

In contrast to the situation in oscines, the activity of TguSINE1 appears to have been longer in suboscines, postdating the divergence between Old World and New World suboscines (i.e., pitta and phoebe in Fig. 3a). This recent, potentially lineage-specific activity coincides with the putative restriction of PittSINEs to Old World suboscines (e.g., *Pitta* spp.), which is further supported by a much lower pairwise distance of PittSINE copies to the consensus (ranging from 0 to 11%, average 6.3%; Additional file 1: Table S3) than in the case of TguSINE1 (Fig. 2b). As mentioned above, the CR1 phylogeny and four diagnostic nucleotides in the CR1-derived SINE tails (cf. Figs. 1c and 2a) indicate that TguSINE1 and PittSINE likely have a common SINE ancestor instead of being derived independently from a CR1-X_Pass LINE. This further suggests that the younger PittSINE emerged from the older TguSINE1 after acquisition of a new tRNA-derived head. Assuming that TguSINE1 and PittSINE were both active on the pitta lineage, we propose that the most plausible mechanism for PittSINE emergence was template switching from TguSINE1 to a nearby tRNA during reverse transcription (Fig. 3b). Slightly less parsimonious alternative explanations for PittSINE emergence might be gene conversion or genomic rearrangement between a TguSINE1 master gene and a tRNA^Asp^ gene, but these remain untestable in the absence of a pitta genome assembly. Template switching has been previously proposed in a wide range of chimeric retroposons (e.g., [44–47]) and appears to be a particularly common opportunity for SINEs to *parasitize* different LINEs via acquisition of new SINE tails [46, 48]. As previously observed for ancient amniote SINEs [49], our data show that template switching may also happen for SINE heads, whereby the acquisition of a new SINE head from a different tRNA and an appropriate upstream sequence close to the insertion site may provide intact and active promoter components for efficient transcription by RNA polymerase III.

## Conclusion

To conclude, we reconstructed the deep phylogenetic relationships of passerines using presence/absence patterns of unusual SINE insertions and other REs. This permitted us to follow the emergence, activity, and extinction of TguSINE1 and PittSINE across the evolution of the most species-rich group of birds. While this SINE activity of ~2000 copies per oscine genome and ~2500 copies per suboscine genome (Additional file 3: Figure S2) was considerably lower than, for example, that in mammals, it nevertheless exemplifies that at least some birds have a more diverse repetitive element landscape than previously anticipated. Furthermore, we note that the activity of TguSINE1 appears to coincide with the evolution of vocal learning during early passerine evolution [13]. Previous evidence suggests that ~4% of birdsong-associated transcripts in the zebra finch brain contain retroposons [5] and it thus remains to be seen whether SINE activity influenced the evolution of, for example, vocal learning in oscine passerines.

## Methods

We identified candidates for presence/absence loci for TguSINE1 and other selected zebra finch retroposons via pairwise alignment of RE loci from zebra finch to orthologous regions in chicken. This was done by comparing and extracting the respective RE-flanking sequences in the UCSC Genome Browser [50], followed by automatic alignment using MAFFT version 6 [51]. In order to find the nine PittSINE marker candidates, we conducted inter-SINE PCR [30] using a single, PittSINE-specific oligonucleotide primer (5′-CTCGTTAGTATAGTGGTGAGTGTC-3′) and standard PCR parameters of ref. [27] with 50 °C annealing temperature. Among the sampled passerines, inter-SINE PCR yielded strong amplification signal only in the pitta (data not shown). Additionally, we identified two TguSINE1 candidate loci in the pitta using a single TguSINE1-specific oligonucleotide primer (5′- CAGTTGGTTAGAGCGTGGTG-3′). All presence/absence screenings were done using oligonucleotide primers binding to conserved RE-flanking regions in chicken/zebra finch alignments (Additional file 1: Table S4), using the touchdown PCR and cloning protocols of ref. [13]. Two recently sequenced species (*Corvus cornix* and *Manacus vitellinus* [6, 52]) were added to reduce missing data in our presence/absence table (Additional file 1: Table S2).

For each presence/absence marker candidate, we first aligned all sequences automatically using MAFFT (E-INS-I option) and then manually inspected these for misalignments. We considered a marker candidate as phylogenetically informative and reliable “*if, in all species sharing this RE, it featured an identical orthologous genomic insertion point (target site), identical RE orientation, identical RE subtype, identical target site duplications (direct repeats, if present) and a clear absence in other species*” [13]. This led to a total of 44 high-quality RE presence/absence markers (Additional file 1: Table S2, Additional file 4).

All maximum likelihood sequence analyses were conducted using RAxML 8.1.11 [53] on the CIPRES Science Gateway [54]. For the CR1 phylogeny, we used the alignment from ref. [20], excluded grebe-specific CR1 elements, and added the CR1-derived tails of TguSINE1 and PittSINE (alignment length 710 bp). For the passerine phylogeny, we removed the RE sequences from our presence/absence alignments and concatenated the remaining RE-flanking sequences into a multilocus alignment (Additional file 4; alignment length 22,410 bp).

Zebra finch TE landscapes were generated from RepeatMasker [55] ‘.align’ files after CpG correction as detailed elsewhere [32]. For PittSINE copies and the PittSINE consensus, Kimura 2-parameter pairwise distances were estimated in MEGA6 ([56]; uniform rates among sites, pairwise deletion of gaps/missing data) after exclusion of CpG sites.

## Additional files

Additional file 1: Tables S1–S4. (PDF 312 kb)

Additional file 2: Majority-rule consensus sequence for PittSINE as reconstructed from our PittSINE-bearing presence/absence patterns. (TXT 155 bytes)

Additional file 3: Figures S1–S2. (PDF 447 kb)

Additional file 4: Fasta-formatted alignments of all RE presence/absence markers. The presented loci are labeled corresponding to the markers listed in Additional file 1: Table S2. The names of transposed elements correspond to those in Repbase. (TXT 805 kb)

Additional file 5: Fasta-formatted multilocus alignment of concatenated RE-flanking sequences used for generating the phylogenetic tree of Fig. 3a. (TXT 660 kb)

## Abbreviations

CR1: Chicken repeat 1
LINE: Long interspersed element
Mb: Million basepairs
MY: Million years
MYA: Million years ago
RE: Retroposed element
RT: Reverse transcriptase
RTBS: Reverse transcriptase binding site
SINE: Short interspersed element
