# Supplementary material for: De-novo emergence of SINE retroposons during the early evolution of passerine birds
Source: Mob DNA. 2017 Dec 14;8:21. doi: 10.1186/s13100-017-0104-1 (PMC5729268; doi:10.1186/s13100-017-0104-1)
Supplement: Supplementary file 1 — (PDF 312 kb) [file 13100_2017_104_MOESM1_ESM.pdf]

Additional file 1: Tables S1–S4.

Table S1: Classification of the RE subfamilies sampled from zebra finch.

Class	Subclass	Superfamily	Family	Subfamily	Described from
I	LINE	CR1	CR1-I	CR1-I_Tgu	taeGut2
I	LINE	CR1	CR1-J	CR1-J3_Pass	taeGut2
I	LINE	CR1	CR1-X	CR1-X1_Pass	taeGut2
I	LTR	ERV1	TguLTR11	TguLTR11n	taeGut2
I	LTR	ERV2	TguERVK9	TguERVK9_LTR2h	taeGut2
I	LTR	ERV3	TguLTR5	TguLTR5c	taeGut2
I	LTR	ERV3	TguLTR5	TguLTR5d	taeGut2
I	SINE	tRNA	TguSINE	TguSINE1	taeGut2

Table S2: Presence/absence matrix of passerine retroposon markers including RE target site duplication (TSD) motifs and location in the taeGut2 assembly of the zebra finch genome.

Marker	*Taeniopygia*	*Petroica*	*Pica*	*Corvus*	*Pomatostomus*	*Malurus*	*Myzomela*	*Climacteris*	*Menura*	*Sayornis*	*Manacus*	*Pitta*	*Acanthisitta*	*Nestor*	RE subfamily	RE orientation	TSD	taeGut2 chromosome	taeGut2 start coordinate	taeGut2 end coordinate
**L-4**	+	?	?	+	?	+	?	?	+	+	+	?	+	-	TguLTR5d	-	GTAAG	2	107.658.311	107.659.269
**L-4**	+	?	?	+	?	+	?	?	+	-	-	?	-	d	TguSINE1	+	?	2	107.658.311	107.659.269
**Pso01**	-	?	?	-	?	?	?	?	-	-	-	+	-	?	PittSINE	-	C	4	43.915.733	43.916.189
**Pso02**	-	?	?	-	?	?	?	?	-	-	-	+	-	?	PittSINE	+	CTATG	5	27.322.271	27.322.008
**Pso03**	+	?	?	+	?	?	?	?	+	+	+	+	-	?	CR1-X1_Pass	+	TTTCT	10	11.861.921	11.861.386
**Pso03**	-	?	?	-	?	?	?	?	-	-	-	+	-	?	PittSINE	-	GAAAT	10	11.861.921	11.861.386
**Pso04**	-	?	?	?	?	?	?	?	-	-	?	+	?	?	PittSINE	+	GGA	3	38.268.171	38.270.277
**Pso08**	-	?	?	-	?	?	?	?	?	-	-	+	-	?	PittSINE	-	CACA	10	1.122.922	1.122.063
**Pso10**	-	?	?	-	?	?	?	?	-	+	+	+	-	?	TguSINE1	+	TG	5	10.716.893	10.716.277
**Pso12**	-	?	?	-	?	?	?	?	-	-	-	+	-	?	CR1-I_Tgu	-	?	7	30.071.845	30.072.221
**Pso13**	-	?	?	-	?	?	?	?	?	-	-	+	-	?	TguSINE1	+	?	5	10.715.853	10.716.265
**Tgu02**	+	?	+	+	?	+	?	+	+	-	-	-	-	-	TguSINE1	+	?	1	10.356.482	10.355.761
**Tgu05**	+	?	+	+	?	?	+	+	+	+	+	?	-	-	TguSINE1	+	A	1	100.547.887	100.547.056
**Tgu07**	+	?	+	+	?	?	+	?	+	-	-	-	-	-	TguSINE1	+	A	1	118.418.987	118.418.291
**Tgu07**	-	?	-	-	?	?	-	?	-	-	-	+	-	-	PittSINE	+	CGATAGTG	1	118.418.987	118.418.291
**Tgu08**	+	+	+	+	?	+	?	+	+	+	+	+	-	-	TguSINE1	+	ATTATT	2	4.459.063	4.459.958
**Tgu09**	+	?	+	+	?	?	?	+	+	?	-	-	-	-	TguSINE1	+	AA	2	19.559.154	19.560.021
**Tgu10**	+	?	+	+	?	?	+	?	+	?	-	+	-	-	TguSINE1	+	?	2	39.949.817	39.949.452
**Tgu13**	+	?	+	+	?	?	?	?	+	-	-	?	-	?	TguSINE1	+	?	2	66.988.522	66.989.422
**Tgu14**	+	?	+	+	+	?	+	+	+	+	?	+	-	-	TguSINE1	+	TGAGGAG	2	75.161.831	75.162.667
**Tgu17**	+	?	+	+	?	?	+	?	+	-	-	?	-	-	CR1-I_Tgu	+	AC	1	20.072.950	20.071.904
**Tgu19**	+	?	+	+	?	?	+	+	+	+	+	?	-	-	CR1-I_Tgu	+	GAATT	2	63.475.805	63.476.911
**Tgu20**	+	?	+	+	?	?	+	?	+	-	-	?	d	-	CR1-I_Tgu	+	?	2	137.203.952	137.204.879
**Tgu21**	+	?	?	+	?	+	?	?	+	+	+	?	+	-	CR1-I_Tgu	+	AAC	3	87.434.090	87.435.182
**Tgu24**	+	?	+	+	?	?	+	+	+	?	+	?	+	-	TguLTR5c	+	AAGAG	4	38.637.563	38.638.584
**Tgu26**	+	?	+	+	?	?	?	+	+	?	-	-	-	?	TguLTR5c	+	AATTA	6	17.528.118	17.526.787
**Tgu27**	+	?	+	+	?	?	?	+	+	-	-	?	-	-	TguLTR5c	+	TGCTC	1A	32.328.895	32.327.600
**Tgu32**	+	-	-	-	?	-	?	-	-	-	-	?	-	-	CR1-J3_Pass	+	?	2	19.478.341	19.479.587
**Tgu33**	+	?	+	+	+	?	?	+	+	+	+	+	-	?	CR1-J3_Pass	+	GA	3	24.954.034	24.952.882
**Tgu34**	+	?	+	+	?	+	?	+	+	-	-	-	-	-	CR1-J3_Pass	+	T	3	32.857.318	32.856.592
**Tgu37**	+	-	-	-	?	?	?	d	?	-	-	-	-	-	TguERVK9_LTR2h	+	CTAGGT	9	6.881.758	6.882.600
**Tgu39**	+	?	+	+	?	?	?	?	+	?	?	-	-	-	TguSINE1	+	TA	2	121.403.718	121.402.809
**Tgu42**	+	?	+	+	?	?	+	+	+	-	-	-	-	-	TguSINE1	+	?	3	11.125.489	11.126.181
**Tgu43**	+	?	+	+	?	+	?	+	+	-	-	-	-	-	TguSINE1	+	AGGATATTAA	3	98.795.454	98.794.809
**Tgu47**	+	+	+	+	?	?	+	+	+	+	+	+	-	-	TguSINE1	+	GAATTT	5	24.046.603	24.045.599
**Tgu48**	+	?	+	+	?	+	?	+	+	+	+	+	+	-	CR1-X1_Pass	+	TATTTA	5	35.475.748	35.475.476
**Tgu51**	+	?	+	+	?	?	+	+	+	+	?	?	-	?	TguSINE1	+	ATTTCAG	6	30.476.496	30.477.321
**Tgu52**	+	?	+	+	?	?	+	+	+	+	+	?	-	?	TguSINE1	+	AT	6	33.164.987	33.165.899
**Tgu58**	+	?	+	+	?	?	+	?	+	?	-	-	-	?	TguSINE1	+	CTT	Z	39.585.532	39.586.342
**Tgu60**	+	?	+	+	?	?	+	+	+	-	-	?	-	d	TguSINE1	+	ACAT	13	15.018.269	15.019.192
**Tgu61**	+	?	+	+	?	+	?	+	?	+	?	+	-	-	CR1-X1_Pass	+	?	15	2.160.370	2.160.694
**Tgu62**	+	-	?	-	?	?	?	?	-	-	-	-	-	-	TguLTR11n	+	TAACC	11	15.647.516	15.648.610
**Tgu65**	+	?	?	+	?	?	?	+	?	?	+	+	+	-	CR1-X1_Pass	+	AAAT	1	1.206.381	1.206.988
**Tgu72**	+	?	?	+	?	?	?	+	+	?	+	?	+	-	CR1-X1_Pass	+	?	2	6.160.531	6.161.492

The character states are '+' (RE presence), '-' (RE absence), or 'd' (unspecific deletion) for each genomic locus. Missing data is indicated by '?'. The TguLTR5d insertion of marker L-4 was first described in Suh, Paus, et al. (2011)

Table S3: Pairwise distances of PittSINE copies to each other and to the consensus sequence.

	Cons.	Pso01	Pso02	Pso03	Pso04	Pso08	Pso15	Tgu07
**Consensus**								
**Pso01**	0.040							
**Pso02**	0.000	0.040						
**Pso03**	0.108	0.138	0.108					
**Pso04**	0.074	0.101	0.074	0.156				
**Pso08**	0.052	0.111	0.052	0.130	0.109			
**Pso15**	0.093	0.127	0.093	0.224	0.168	0.187		
**Tgu07**	0.072	0.121	0.072	0.190	0.131	0.138	0.000	

Table S4: Oligonucleotide primer sequences.

Marker	forward primer (5'-3')	reverse primer (5'-3')
**L-4**	CAGTTGTGTGAGCTTTCCTTG	CAGAAGAAAAATTGTATGATAATGG
	GCTTTCCTTGTTGATATGGTTG	CTGTTTCTTGCTTATTGTGTTGG
**Pso01**	AAACCAGAGTATCCCACTTGC	CATTAGAAATCAGCTCAAACAGTG
	AGAGTGCCACAGATGCCTG	CTCATTTTTCCATTTCCAGC
**Pso02**	ACTTGGGATGAGAGGAGAGC	ATAAATTAGGCAGGCAAAGC
	GGATGAGAGGAGAGCTTGC	GCTATTTGTACGAAGATATGTAATGC
**Pso03**	GTCACAACGCAGTTCTGATG	CCTTAGAAATGGAGAAGCCTG
	GGGTATTTACTACTTTTGTTTGTGC	GCTGTGTGTAGGATCTGTTGC
**Pso04**	CTCTCTGAAGTGGACTGTGTCTC	AGCTGAGGGCACATAGTAGC
	TCTGAAGTGGACTGTGTCTCATC	TGAGGGCACATAGTAGCTGC
**Pso08**	ACTCTGTCCTGACCTTATCCAG	CAGAACAAAGCCAAGCAATG
**Pso10**	TGCTGGCTTTGAATGACAG	TGCCACATTTCTTAGTGATGG
	GGCTTTGAATGACAGCTACTG	TGACCCCCATATCAGGAGC
**Pso12**	GAAGAAACCACAGCTTCAAGG	GTCATGTTCAGACAGGCAGG
	AGAAACCACAGCTTCAAGGG	ATGTTCAGACAGGCAGGATG
**Pso13**	CACGAGTTTTAGAGGCATAACC	GCGAAGTATTTACCATCACTAAG
	CAGACTGTTCCACCAAGCAG	
	AGCTGAGAAAGCACCAAAGTC	GAAATGTAGCATCCAGTGAGAAG
**Tgu02**	TGTTCAACATTCATTCCTTGG	AAATCTCAGAGGAGCTTCGTAC
	GCCAGTAAGCTCAGTTTTTCAG	CCATAGGAATATCAAAGAACCTG
**Tgu05**	GCAGGAAATGCCTTTCTG	CACTGTTCTCCATCATCTTCAG
	GAAATGCCTTTCTGGTTTTG	CTTTCTAAACTTGGGTTCTATGC
**Tgu07**	AAGGAGAGGTCAGTGAATTGG	TCATCAGGGATCTGACTTGC
	ACAAAGTATTTGAGAGCCAGC	ATGACCCAGCCCTGTCAG
**Tgu08**	AACCAACAACTCTAAAAGCCAC	GCCCAAAGATGACATCGTG
	GGGAAGGAGGAATCTGAATAC	TGGACAGAGTTAAGATGGAAGAC
**Tgu09**	CAATTAGTGAGTTATAGCCACACAC	CATGACCAACAGTAACCATCAG
	ACACTGCAATACATGTGATAAGTC	TCAGACTTCTAACAGCAAGAGC
**Tgu10**	TCATATCAATTCAAGGCAAGG	ATCACAAGGCTGGATGAGC
	AGGTTCCCTGTTCGTTACTG	GGATGAGCTTGCTGTTGC
**Tgu13**	GCCACCACAACATCCCAC	GGAGAGGGTGACTATTGTCTGTG
	GCAGAAGTGGAGATTTTAAGC	GGGTGACTATTGTCTGTGAGG
**Tgu14**	TTCGTAGACTTCACAGAAATCAC	CCTTTCACTGCTGCTACCAG
	CCTGCCTATTTAGTGTAGTTCAG	CTGCTACCAGTGCAAAAGC
**Tgu17**	ATCTGCCAAGACAGCAAGTC	CCTGATCCAATTCAATCCAG
	AGACCAAGTAGAAAGATTCCCTC	CACACACAAAGACTAAACAACTGG
**Tgu19**	AACCTCGACTTCCAGATGG	GAAGAAATAACGCTCACATCAG
	TACAAAAGGAAGAGCGATGG	CAGGTCCTCTTCAGATTGATG

Table S4: Continued.

**Tgu20**	TTGCTTGGCACCAGACAC	AAACAGGTGGTCCTCTGAGC
	GCCTGACATTATCTTTCCCTG	CAAGGCAAATGCTGATGTTC
**Tgu21**	AGGATCAGTGGAAATTGGTTC	CTTTTACAGAAGCCATAAGTGC
	GCCCTGTGACAGCACTAATG	CACAGTTCAGACCTTCAAATCC
**Tgu24**	AGGAGTAATAGCCTGTGGAAGA	CTGGTATTGAGACAGACAGTGG
	GAGTAATAGCCTGTGGAAGAGAG	ACAGACAGTGGATCAGAAGAAAC
**Tgu26**	GGAGTAGCAATGTACCCATAGAC	TGAAGTATCAAGTCCTGCCTC
	CATAGACATCAATAAAGGAAGAGG	AGCCCTGTCTTCTAAATCTCAG
**Tgu27**	GTGGAAAATCATTGAGGACTG	GCAGTTCAAGGGAATAGTAACAG
	AGCCAGTAGGGAGCATCAG	AGTTCAAGGGAATAGTAACAGAAG
**Tgu32**	GGGAATTTGATCCAGCTATTG	GATACTTACCCCATGCTTTACTG
**Tgu33**	CCTTACCATCTCCTAATGACCTC	CCATCATCTTTTTCAGAGCATC
**Tgu34**	ATAGGTGGTGATGATGATTGG	TTCCAGTGTCTGCCATCTG
	CCTGGAGCTGGCAAGACTAC	ATCTGACGCTTAAAACCTGC
**Tgu37**	ATTGAGGGAGTCAAAGGAGC	CATCTCAGTACATCTGCTGTGC
	GTCAAAGGAGCCAAGAACTTC	CAGAAAGGGTGCTTTGTCAG
**Tgu39**	CTTCTGTTCCTCAAACCCAC	CTTGCCTATTCTTCCTTTCAG
	TTCCTCAAACCCACCAGAC	CTTCCTTTCAGGTGTTTTGC
**Tgu42**	AAATTAAGCCTCTAGTCTGTTCTG	CCTCCCTTCAGGTTTCCAG
	ATTAAGCCTCTAGTCTGTTCTGG	CCTTCAGGTTTCCAGTGAAC
**Tgu43**	GGAAGCCATCACAACACTTG	GTTTATCAGACATACGTCAATGC
**Tgu47**	CTGCTTGCTTCCCTCTCTG	ACCTCTGTTTCTACATCAGTGTAAG
	CTTGCTTCCCTCTCTGTTTC	CCCAGCTAGACAGGAAAGATG
**Tgu48**	TTGAGATGTAGAGCTTGACACTAAC	AGAGATTCTGCCTCTATTCTTTG
**Tgu51**	ATTCGCAGTCCAGATTTCC	TTGACAGGAGGGATAACAACC
	CGCAGTCCAGATTTCCTG	ATCAAACAAGATCCTGTGAGC
**Tgu52**	TCACCACAAAGCAGATGATG	GGCAGAGAAATCCTTCCTG
	CACCACAAAGCAGATGATGC	AGGTTGGATATCAGGAAAAGG
**Tgu58**	TCTCCTCTGCTGCTGGTTC	GCCCAGATTGCTAATGAAAG
	TCCTCTGCTGCTGGTTCTAG	CCCAGGGTAAATACAGATGC
**Tgu60**	CCAGGCAGAAGGTGAACTG	GTCTTGGACTTTTGTTTTGACC
	CAGTGCAGGAAGTTGGAGTG	TGACCAGATTTGCTCCCTG
**Tgu61**	ATCAAGAAGCTCAGCACGAC	GGGATTGACAGCCTCAGAG
	ACGACCAACAAAAACGAGAC	GGATTGACAGCCTCAGAGC
**Tgu62**	CTGGGAGTGTTAATGGTGAGAG	GATTTATAGTGCCCTTCATAAGC
	GAGTGTTAATGGTGAGAGTGTGC	ATAAGCGTAAAGGTGTTCTGC
**Tgu65**	GGAGCAGTCACAGTCTTCAGG	TGTGTGTTTCACAGTCCATAGC
**Tgu72**	ATATTTGCTACTAGGTTGCTACG	ATTTACCCATTTGCCTGAAG
