# Supplementary material for: De-novo emergence of SINE retroposons during the early evolution of passerine birds
Source: Mob DNA. 2017 Dec 14;8:21. doi: 10.1186/s13100-017-0104-1 (PMC5729268; doi:10.1186/s13100-017-0104-1)
Supplement: Supplementary file 3 — (PDF 447 kb) [file 13100_2017_104_MOESM3_ESM.pdf]

Additional file 3: Figures S1–S2.

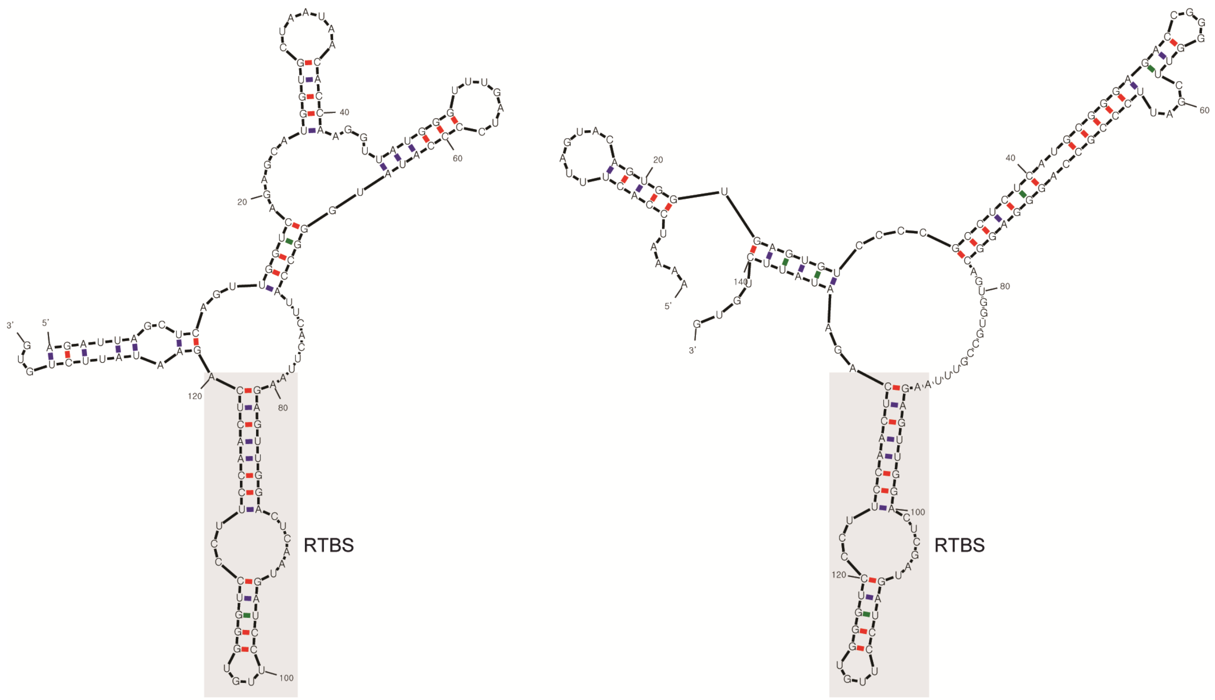

TguSINE1 (dG = -30.99 [Initially -38.70]) **PittSINE** (dG = -47.47 [Initially -51.50])

Figure S1: Predictions of secondary structures of TguSINE1 and PittSINE using mfold [57].

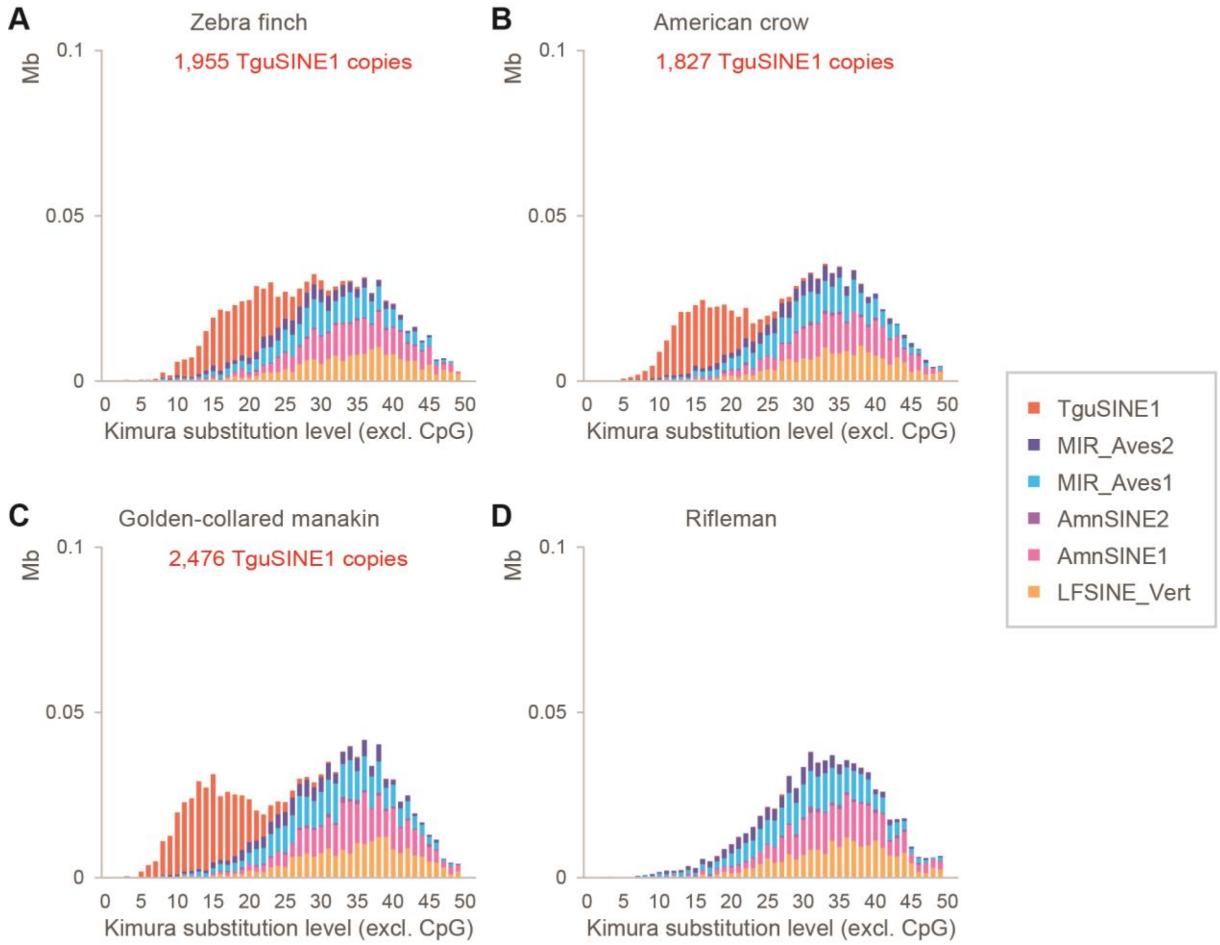

Figure S2: Landscape plot of temporal and quantitative SINE activity in representative genome assemblies of passerine birds. The genome assemblies [6] of oscines (*Taeniopygia guttata*, panel A; *Corvus brachyrhynchos*, panel B), suboscines (*Manacus vitellinus*, panel C), and the rifleman (*Acanthisitta chloris*, panel D) mostly contain ancient CORE-SINEs present in all amniotes [7]. TguSINE1 copy numbers were estimated by counting only those RepeatMasker hits that contained at least 10 bp of both the SINE head and tail, respectively.
